# Validation of candidate genes putatively associated with resistance to SCMV and MDMV in maize (*Zea mays *L.) by expression profiling

**DOI:** 10.1186/1471-2229-9-15

**Published:** 2009-02-02

**Authors:** Anna Użarowska, Giuseppe Dionisio, Barbara Sarholz, Hans-Peter Piepho, Mingliang Xu, Christina Rønn Ingvardsen, Gerhard Wenzel, Thomas Lübberstedt

**Affiliations:** 1 Department of Plant Breeding, Technical University of Munich, Am Hochanger 2, 85350, Freising, Germany; 2 Faculty of Agricultural Sciences, University of Aarhus, Department of Genetics and Biotechnology, Research Centre Flakkebjerg, Slagelse, DK-4200, Denmark; 3 General Motors Powertrain Germany GmbH, 65423, Rüsselsheim, Germany; 4 Department of Bioinformatics, University of Hohenheim, Fruwirthstrasse 23, 70593, Stuttgart, Germany; 5 National Maize Improvement Center of China, China Agricultural University, 2 West Yuanmingyuan Road, Beijing, 100094, PR China; 6 Department of Agriculture and Ecology, Faculty of Life Sciences, University of Copenhagen; 7 Department of Agronomy, Iowa State University, 1204 Agronomy Hall, 50011 Ames, Iowa, USA

## Abstract

**Background:**

The potyviruses sugarcane mosaic virus (SCMV) and maize dwarf mosaic virus (MDMV) are major pathogens of maize worldwide. Two loci, *Scmv1 *and *Scmv2*, have ealier been shown to confer complete resistance to SCMV. Custom-made microarrays containing previously identified SCMV resistance candidate genes and resistance gene analogs were utilised to investigate and validate gene expression and expression patterns of isogenic lines under pathogen infection in order to obtain information about the molecular mechanisms involved in maize-potyvirus interactions.

**Results:**

By employing time course microarray experiments we identified 68 significantly differentially expressed sequences within the different time points. The majority of differentially expressed genes differed between the near-isogenic line carrying *Scmv1 *resistance locus at chromosome 6 and the other isogenic lines. Most differentially expressed genes in the SCMV experiment (75%) were identified one hour after virus inoculation, and about one quarter at multiple time points. Furthermore, most of the identified mapped genes were localised outside the *Scmv *QTL regions. Annotation revealed differential expression of promising pathogenesis-related candidate genes, validated by qRT-PCR, coding for metallothionein-like protein, S-adenosylmethionine synthetase, germin-like protein or 26S ribosomal RNA.

**Conclusion:**

Our study identified putative candidate genes and gene expression patterns related to resistance to SCMV. Moreover, our findings support the effectiveness and reliability of the combination of different expression profiling approaches for the identification and validation of candidate genes. Genes identified in this study represent possible future targets for manipulation of SCMV resistance in maize.

## Background

SCMV and MDMV are positive-sense single strand RNA potyviruses that cause significant yield loss in susceptible genotypes of maize, sugarcane, and sorghum [1,2]. SCMV is notably harmful in Europe and China, MDMV in the southern US Corn Belt [3]. Both closely related potyviruses are transmitted in a non-persistent manner by aphids mainly to members of the *Poaceae *family [4]. Disease symptoms are mosaic, chlorosis, leaf reddening, necrosis, and stunting [2,5]. Both viruses spread systemically and particularly fast in young susceptible plants [6].

Out of 122 early-maturing maize dent inbred lines investigated by Kuntze et al. [7], three (D21, D32, and FAP1360A) were found to be completely resistant to SCMV, MDMV, JGMV, and SrMV, both in field and greenhouse experiments. Depending on the population used, one to five genes were assumed to be required for complete SCMV or MDMV resistance [3,8-11]. Two major SCMV resistance genes, *Scmv1 *and *Scmv2 *were mapped to chromosomes 6S and 3L, respectively, by utilising QTL mapping and bulked segregant analysis (BSA) [1,12-14]. Additional three minor QTL were identified on chromosomes 1, 5, and 10 [1]. Presence of resistance alleles at both loci, *Scmv1 *and *Scmv2*, is crucial for complete SCMV resistance. *Scmv1 *suppresses symptoms at all developmental stages, *Scmv2 *at later stages of infection [1,15]. One major MDMV resistance gene (*Mdmv1*) mapped to the same region of chromosome 6S as *Scmv1*. So far, it is not clear, whether or not *Mdm1 *and *Scmv1 *are the same or closely linked genes. The *Scmv1*/*Mdmv1 *chromosome region contains a cluster of resistance gene analogues [4,16], making both possibilities equally likely.

Expression profiling based on microarrays allows generation of global gene expression patterns for any developmental stage, tissue type, or environmental factor [17]. The method has previously been successfully applied for identification of SCMV resistance candidate genes in maize [18,19]. Suppression Subtractive Hybridization (SSH) and unigene-microarrays identified a subset of differentially expressed genes in response to SCMV infection, the majority of which related to cell rescue and defence, signal transduction, and transcription categories. Moreover, some of the genes identified co-localised with SCMV resistance genes *Scmv1 *and *Scmv2*. Thus, expression profiling seems to be an appropriate tool to study host-resistance responses and to detect pathogenesis-related genes.

The objectives of this study were to a) compare expression profiles of four near-isogenic lines after infection with SCMV or MDMV: F7 ^SS/SS^, F7 ^RR/RR^, F7 ^SS/RR ^and F7 ^RR/SS^, carrying fixed susceptibility (S) or resistance alleles (R) at the *Scmv2 *and *Scmv1 *locus, respectively; b) compare expression patterns between time points, from the time of mechanical inoculation until 24 hours after inoculation; c) compare expression profiles for infection with two different viruses; d) investigate the potential and reliability of the combination of two expression profiling technologies, such as microarrays and quantitative real time RT-PCR in the identification of validated differentially expressed genes for SCMV/MDMV resistance; e) identify candidate genes for *Scmv1 *and *Scmv2 *that could potentially be utilised in breeding for virus resistance; and f) relate the findings of this study to previous SCMV experiments.

## Results

### SCMV/MDMV phenotype analysis

Twenty-two out of 32 and 27 out of 32 F7 ^SS/SS ^plants showed visible disease symptoms two weeks after inoculation with SCMV and MDMV, respectively. Symptom appearance was not tested for additional weeks, due to previous experience with the potyvirus pathosystem, where 100% infected plants of the susceptible genotype occur at later stages (three to seven weeks). The occurance of symptoms in the other three near-isogenic lines depends on the presence of resistance loci and has been thoroughly tested before [1,20].

### cDNA microarray-based expression profiling

#### SCMV experiment: within-time-point analysis

4578 observations for each of the five time points and the mock control were collected for pair-wise comparisons of near-isogenic genotypes, giving altogether 27468 observations across all time points. 65 sequences showed significant differential expression within time points and pair-wise contrasts at a FDR level of p ≤ 0.05 (including double-counting of sequences differentially expressed at different time points) (see Additional file 1). In total 28 different genes showed differential expression across pair-wise contrasts and time points, representing 3.7% of 762 printed genes (excluding controls), with only 3 genes being expressed in the mock control experiment (Table 1).

**Table 1:** 28 significantly differentially expressed genes after SCMV inoculation within time points.

**EST**	**Genotype** ** F7 ^SS/SS ^** **- F7 ^RR/RR^**	**Genotype** ** F7 ^SS/SS ^** **- F7^SS/RR^**	**Genotype** ** F7 ^SS/SS ^** **- F7^RR/SS^**	**Genotype** ** F7 ^RR/RR ^** **-F7^SS/RR^**	**Genotype** ** F7 ^RR/RR ^** **- F7^RR/SS^**	**Genotype** ** F7 ^SS/RR ^** **- F7^RR/SS^**
*605018B03.x1*	2,3,9			2,3,9	2	
*605018B04.x1*	2,3,5,9		3	2,3,5,9	2,9	
*606007B06.x1*	3		3			
*606021F11.x2*			5			
*614013G06.x1*		3				
*614044F12.x4*	1		1	4		4
*945031C10.X1*				2		
*949062B09.y1*					2	
*MEST12-E11.T3*		4		2,4		4
*MEST19-G10.T3*	2					
*MEST22-A03.T3*						9
*MEST24-E10.T3*	2			2		
*MEST24-G11.T3*	2			2		
*MEST40-B08.T3*	2				2,5	
*MEST40-G05.T3*				2		
*MEST41-B03.T3*				4		
*MEST63-E12.T3*				2		
*MEST67-A07.T3*					3,4	4
*MEST82-F04.T3*	2			2	2	
*MEST333-H11.T3*				2		
*Zm06_09h07_R*		4		2	2	
*PAC000000001182*				2		
*946126A02.y1*				2	2	
*1091032B12.y1 a*			4			
*1091032B12.y1 b*				2	2	
*za72g09.b50*				2		
*946063C12.y1*					5	
*exon 1 (eIF3E barley gene)*				2	2	

Total	9	3	5	18	11	4

Differential expression presented for a given genotype pair and time point.

**Figure 1:**
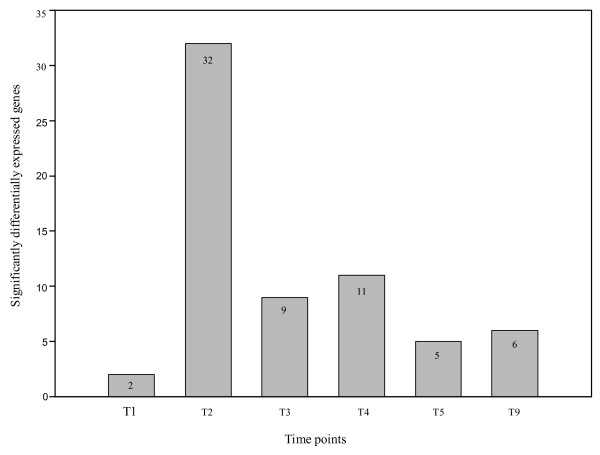
**The number of significantly differentially expressed genes in the SCMV experiment over six time points (including mock control as T9)**.

The majority of significantly differentially expressed sequences was identified for time point T2 (0.69% of the 4578 observations of all differential genes for a given time point), followed by T4 (0.24%), T3 (0.19%), T9 (0.13%), T5 (0.10%), and T1 (0.04%) (Figure 1). The number of expressed sequences was significantly different between T2 and all other time points at a significance level of 1%, and between T1 and T4 at a significance level of 5% (McNemar test).

**Figure 2:**
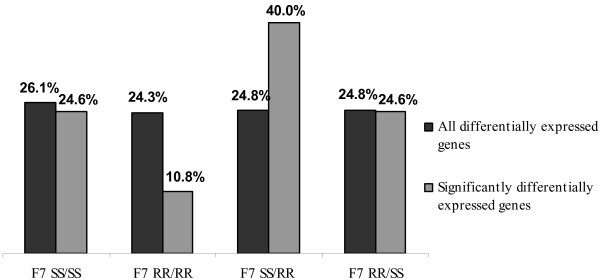
**Percentages of genes up-regulated in near-isogenic lines (within-time-point SCMV experiment)**.

**Figure 3:**
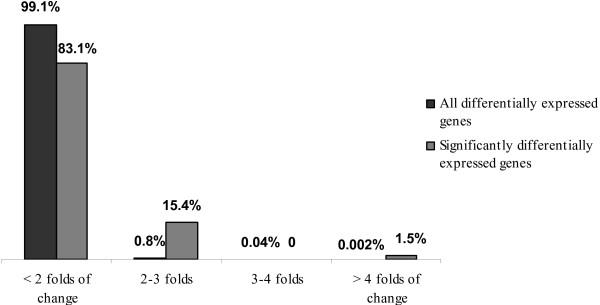
**Percentages of genes expressed in the within-time-point SCMV experiment based on their folds of change**.

In the whole set of 27468 observations a similar distribution of up-regulated genes was found for all four near-isogenic genotypes. When considering only significantly differentially expressed genes, the majority of genes were up-regulated in genotype F7 ^SS/RR^, carrying the *Scmv1 *resistance locus (Figure 2). Except for 16.9% of genes, the folds of change of significantly differentially expressed genes were below 2-fold (Figure 3).

Six pair-wise genotype-contrasts were considered. Most significantly differentially expressed genes summarised over all time points were found between F7 ^RR/RR ^- F7 ^SS/RR ^(18 genes), followed by F7 ^RR/RR ^- F7 ^RR/SS ^(11 genes), and F7 ^SS/SS ^- F7 ^RR/RR ^(9 genes) (Table 1). Out of the 28 different genes showing differential expression, 13 were in common among two pair-wise contrasts, 7 among three and 2 among four pair-wise contrasts. None of the genes showed common differential expression among five or all six pair-wise comparisons.

Two genes (*605018B04.x1 *and *605018B03.x1*) were most commonly significantly differentially expressed within time points and for different genotype pairs, 12 and 7 times respectively (Table 1, see Additional file 1).

#### SCMV experiment: between-time-point analysis

**Figure 4:**
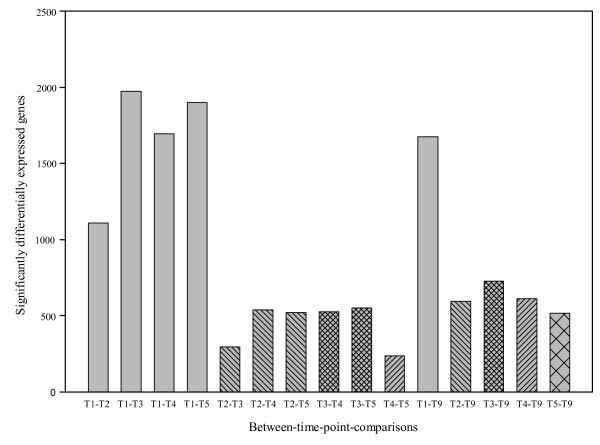
**Significantly differentially expressed genes in comparisons between time points across the four genotypes (SCMV experiment)**.

14070 out of 45780 observations showed significant differential expression between time points at the level of p ≤ 0.05. Most significantly differentially expressed genes were found in comparisons of time point T1 with all other time points (Figure 4).

**Figure 5:**
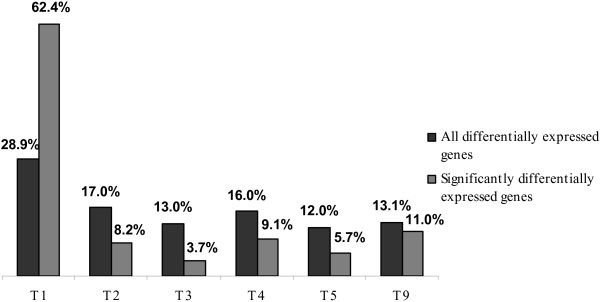
**Percentages of genes up-regulated in single time points (between-time-point SCMV experiment)**.

The majority of genes were up-regulated in T1, with significantly differentially expressed genes surpassing all observations in this time point by more than double (Figure 5).

All 28 genes from the within-time-point analysis were also significantly differentially expressed in the between-time-point analysis (see Additional file 2).

#### Comparison to previous SCMV studies

In order to maximise the chance to identify putative candidate genes involved in resistance to SCMV and MDMV, pre-selected SCMV candidate genes were spotted on the array utilised for this study. Shi et al. [18,19], in their studies on SCMV infected maize reported 302 and 497 differentially expressed genes when utilising macro- and microarray approaches, respectively. 451 of those genes were successfully amplified and included in our microarray experiments. The remaining about 40% of genes included on our custom microarray comprised resistance candidate genes and resistance gene analogues. For the 65 sequences, differentially expressed within-time points, 80% were derived from the pre-selected genes. When considering redundancy of detecting the same gene within time points repeatedly, 4.4% (21 genes) of the pre-selected genes, but only 1.6% resistance gene analogues (7 genes) showed differential expression.

### Ontology description of genes differentially expressed in the SCMV experiment

Maize molecular function GO assignment  was performed for 20 significantly differentially expressed ESTs with available annotations from the within-time-point experiment. Since more than one biological function can be assigned, 30 GO hits with 19 GO terms were obtained. Out of these, six each were assigned to catalytic activity (homology to sphingolipid/alcohol dehydrogenase and phosphatidic acid phosphatase) and molecular function unknown (homology to S-like RNase or 26S ribosomal RNA), respectively, four to transporter activity (homology to cytochrome c and sphingolipid/alcohol dehydrogenase) and three to binding activity (homology to metallothionein-like protein and sphingolipid/alcohol dehydrogenase) (see Additional file 1). Moreover, three homologous genes (pathogenesis-related protein, alcohol dehydrogenase, and glutathione S-transferase) have been previously determined as pathogenesis-related genes [21].

### Genetic map positions

For 7 out of 20 ESTs map positions were determined *in silico *using the Maize GDB , while seven of the RGA sequences were mapped beforehand (Table 2). For some genes, more than one map position was available based on mapping experiments or various blast applications (EST, GSS, EST TUG, maize nucleotide). The majority of genes (six) was located on chromosome 8, in a continuous bin 8.04 – 8.06, whereas three genes each were located on chromosomes 10 (bin 10.04) and 1 (bin 1.02 and 1.06 – 1.07), respectively. Furthermore, two genes were assigned to chromosome 6 (bins 6.00 and 6.07), carrying the *Scmv1 *resistance gene, and another two to chromosome 3 (bin 3.04, 3.09), carrying the *Scmv2 *gene.

**Table 2:** Map positions for SCMV identified significantly differentially expressed ESTs and RGAs (sequences from CAU collection)

**EST**	**Map positions**
*605018B03.x1*	bin 1 (1.02)^3^, bin 6 (6.00)^4^, bin 7 (7.02)^3^
*606021F11.x2*	bin 3 (3.09)^2^
*614044F12.x4*	bin 8 (8.04)^1,2^
*MEST19-G10.T3*	bin 10 (10.04)^1,3^
*MEST40-G05.T3*	bin 8 (8.06)^2^
*MEST67-A07.T3*	bin 1 (1.06)^3^
*MEST82-F04.T3*	bin 10 (10.04^)1,3^
*Zm06_09h07_R*	1.07; 2.04; 2.09; 4.08; 10.04
*PAC000000001182*	6.07
*946126A02.y1*	8.05; 8.06
*1091032B12.y1 a*	8.05; 8.06
*1091032B12.y1 b*	8.05; 8.06
*za72g09.b50*	3.04
*946063C12.y1*	8.05; 8.06

^1 ^EST, ^2 ^GSS, ^3 ^EST TUG, ^4 ^Maize Nucleotide

#### MDMV experiment: within-time- point analysis

Only two genes were significantly differentially expressed in the MDMV experiment at a FDR level of p ≤ 0.05 within time points. The two genes were significantly differentially expressed at two different time points and all were up-regulated in F7 ^SS/SS^. One of the two genes (*605018B04.x1*) was also significantly differentially expressed within time points in the SCMV experiment. The fold of change did not exceed 3-fold for all three significant gene × time point combinations (data not shown).

GO description for *605018B04.x1 *was binding activity (metallothionein-like protein), whereas no GO assignment but homology to a solanesyl diphosphate synthase was found for the second gene (*947026D04.x1*) .

#### MDMV experiment: between-time-point analysis

Forty-six percent out of 7260 observations showed significant differential expression between time points at a FDR p level ≤ 0.05. The majority of up-regulated differentially and significantly differentially expressed genes were found for T1 (see Additional file 3). Distribution of genes regarding their fold changes is shown in Additional file 4.

The two genes (*605018B04.x1*, *947026D04.x1*) identified in the within-time-point analysis as significantly differentially expressed were also significantly differentially expressed in the between-time-point analysis (see Additional file 5).

### SCMV experiment: quantitative RT-PCR

Six out of the 65 consistently differentially expressed sequences from microarray experiments were selected for validation by qRT-PCR based on their map position, expression pattern, fold of change in microarray experiments, or sequence homology related to resistance response genes. These included genes expressing a metallothionein-like protein, 26S ribosomal RNA, 14-3-3-like protein GF14-6, two genes for S-adenosylmethionine synthetase 1, and germin-like protein 4 (Table 3). An endogenous maize actin gene was used as a reference in this experiment. Coefficients of determination (R^2^) for reference and target genes were between 0.94 and 0.99, confirming good quality of standard curves. PCR efficiencies for target and reference genes ranged from 1.0 to 1.4, except of germin-like protein deviating from the standard PCR efficiency for target gene up to E = 3.6 (Table 4).

**Table 3:** Sequence homologies for selected SCMV differentially expressed genes

**Gene ID**	**Time point**	**Genotype**	**Microarrays** **FDR p-value**	**Microarrays** **fold of change**	**GO**	**TIGR description (homology)**	**Map position (bin)**
*605018B03.x1*	T2	F7 ^RR/RR^ F7 ^SS/RR^	2.317E-07	2.1	Molecular function unknown	gb|AF036494.1|AF036494 Eucryphia lucida large subunit 26S ribosomal RNA gene, partial sequence, partial (52%)	1.02/6.0/7.02
*605018B04.x1*	T2	F7 ^RR/RR^ F7 ^SS/RR^	0	2.6	Binding	UP|Q5U7K6_9POAL (Q5U7K6) Metallothionein-like protein, partial (94%)	-
*946126A02.y1*	T2	F7 ^RR/RR^ F7 ^RR/SS^	0.0140382	1.7	-	UP|METK_ORYSA (P46611) S-adenosylmethionine synthetase 1 (Methionine adenosyltransferase 1) (AdoMet synthetase 1), complete	8.05
*Zm06_09h07_R*	T2	F7 ^RR/RR^ F7 ^SS/RR^	1.085E-05	1.6	-	UP|14331_MAIZE (P49106) 14-3-3-like protein GF14-6, complete	1.07
*946063C12.y1*	T5	F7 ^RR/RR^ F7 ^RR/SS^	0.04668	2.0	-	UP|METK_ORYSA (P46611) S-adenosylmethionine synthetase 1 (Methionine adenosyltransferase 1) (AdoMet synthetase 1), complete	8.05
*za72g09.b50*	T2	F7 ^RR/RR^ F7 ^SS/RR^	0.033618	1.4	-	similar to UP|O49000_ORYSA (O49000) Germin-like protein 4, complete	3.04

**Table 4:** Comparison of SCMV microarray and qRT-PCR results

	**Fold of change**		**qRT-PCR performance**	
	
**Target gene**	**Microarrays: four biological replications (average) ± SE**	**qRT: four biological replications (average) ± SE**	**Coefficients of determination (R) target/reference**	**PCR efficiencies target/reference**
*605018B03.x1*	2.1 (± 0.16)	1.6 (± 0.18)	0.99/0.99	1.1/1.2
*605018B04.x1*	2.6 (± 0.15)	89.2 (± 14.82)	0.99/0.98	1.1/1.4
*946126A02.y1*	1.7 (± 0.18)	1.2 (± 0.18)	0.98/0.98	1.0/1.1
*Zm06_09h07_R*	1.6 (± 0.12)	1.4 (± 0.13)	0.99/0.99	1.1/1.1
*946063C12.y1*	2.0 (± 0.26)	2.7 (± 0.37)	0.97/0.99	1.1/1.3
*za72g09.b50*	1.4 (± 0.13)	2.7 (± 0.19)	0.94/0.99	3.6/1.1

Differential expression of the metallothionein-like protein homologue (*605018B04.x1*) was validated by qRT-PCR with a fold change of 89.2 (average from four biological replications) as compared to 2.6 (p = 0.0) fold from microarray experiments. The S-adenosylmethionine synthetase 1 gene (*946063C12.y1*) and germin-like protein 4 (*za72g09.b50*) were validated with a fold of 2.7, as compared to 2.0 and 1.4 fold from microarrays, respectively. The putative 26S ribosomal RNA gene (*605018B03.x1*) and the S-adenosylmethionine synthetase 1 gene (*946126A02.y1*) were not validated when averaging four biological replications (1.6 and 1.2 fold, respectively), but had significant fold of changes in one of the four replications (data for separate replications not shown). The 14-3-3- like protein GF14-6 (*Zm06_09h07_R*) was not validated by qRT in any of the four biological replications. However, the fold value for three replications ranged from 1.6 to 1.7 fold.

## Discussion

### Validation and reliability of data

#### Comparison to previously published data

The purpose of this study was to identify and validate genes involved in resistance response to SCMV and MDMV. In previous SCMV experiments [18,19], a set of candidate genes was identified to show significant differential expression between near-isogenic SCMV resistant and susceptible inbred lines. These genes, together with resistance gene analogs (RGAs) were spotted on our cDNA SCMV array. Twice as many genes based on earlier studies showed differential expression as compared to RGAs, indicating usefulness of pre-selection and reliability of microarray approach.

#### Comparison of SCMV and MDMV experiments

The MDMV experiment was set up to compare response of isogenic lines containing *Scmv1 *and/or *Scmv2 *regions from the resistant FAP1360A inbred line to related but different viruses. Comparative studies of related viruses displaying common symptoms in the same host offer an opportunity to link changes in global gene expression to specific symptoms and to identify common genes involved in resistance responses [22]. It was assumed in the experimental design that F7 ^RR/RR ^demonstrates full resistance to both SCMV and MDMV [20]. However, the finding of only very few significantly differentially expressed genes within time points in the MDMV compared to the SCMV experiment supports the reliability of our SCMV results, because the MDMV experiment compared only susceptible, while the SCMV experiment resistant and susceptible isogenic genotypes.

#### Comparison of array and qRT data

One of the most important issues after performing microarray experiments is validation of their results. Preferentially significantly differentially expressed genes within time points for comparisons of F7 ^RR/RR ^with F7 ^SS/RR ^or F7 ^RR/SS ^were considered. The 26S ribosomal RNA gene was chosen because of its putative map position on chromosome 6 and high fold change, whereas the gene putatively expressing a metallothionein-like protein was selected due to its high fold change and differential expression at all time points. The gene putatively expressing a 14-3-3-like protein GF14-6 and the S-adenosylmethionine synthetase 1 were selected due to their expression pattern, and the germin-like protein 4 due to its map location on chromosome 3.

The genes coding for metallothionein-like protein, S-adenosylmethionine synthetase 1, and germin-like protein 4 were confirmed by qRT experiments to be differentially expressed at significant levels. The germin-like protein showed very high PCR efficiencies, a likely result of template quantity, presence of inhibitor or high RNA purity (data not shown). Differential expression of the 26S ribosomal RNA gene and the second S-adenosylmethionine synthetase 1 gene was confirmed, but only in one of the four biological replications. Possible reasons of these findings are a) false positive results in microarray experiments, b) pooled performance (biological and technical replications) of array data as compared to analysis of pooled technical but separate biological replications for qRT, or c) low fold change values from microarray experiments (despite of significance), which might be difficult to reproduce by other methods if close to the significance threshold. Similar findings were reported by Czechowski et al. [23] and Dallas et al. [24], who indicated that only genes with higher expression levels from microarray experiments (> 1.5 folds of change) are likely to be validated by qRT-PCR.

#### Molecular mechanisms of plant response reaction to virus invasion

Candidate genes identified in this study were annotated according to Maize Gene Ontology Assignment to three major groups: genes encoding catalytic activity (oxidoreductase and hydrolase activities), genes with molecular function unknown, and genes encoding transporter activity (electron transporter activity). Catalysis provides chemical energy required for maintenance of living cells and is of particular importance for the plant while delimiting pathogen invasion. Catalytic activity of oxidoreductase (redox reactions) has been speculated to be crucial for the survival of host plants, since it is required for energy transduction, operation of many anabolic and catabolic pathways, nutrient assimilation, and for defence against disease organisms [25].

One of the functions of redox activity is the formation of hydrogen peroxide (H_2_O_2_), which generates a response to pathogen attack and enhances cells lignification and/or structural protein polymerization, thus producing a mechanical barrier for the invader that have not yet entered the symplasm. A rapid oxidative burst of H_2_O_2 _production has been previously reported as a result of pathogen invasion [26-29]. Moreover, Apostol et al. [30] speculated that H_2_O_2 _production may be part of a signal transduction mechanism for coordination of cellular defences. Furthermore, redox reactions depend on electron supply and active oxygen species. Therefore, electron transfer activity is thought to be unseparable from defence mechanisms [31].

Additional comparative gene annotation was based on results obtained by Whitham et al. [21] on *Arabidopsis *infected with five distinct viruses, including a mosaic potyvirus. Three homologous genes (pathogenesis-related protein, alcohol dehydrogenase and glutathione S-transferase) were identified in the classes cell rescue, defence, death, and ageing of *Arabidopsis*, thus indicating the reliability of microarray technology for detection of pathogenesis-related genes.

#### Association of map positions of differentially expressed candidate genes with Scmv1 and Scmv2

A virus resistance gene needs to be expressed before pathogen invasion, in order to enable a rapid response after infection. Its expression may increase further after virus attack. In previous QTL experiments [1], *Scmv1 *(as QTL) was detectable at each scoring time point after inoculation, whereas *Scmv2 *became first detectable and induced at later scoring stages. Assuming that both *Scmv1 *and *Scmv2 *are single genes, clustering of differentially expressed genes in the *Scmv1 *and *Scmv2 *genomes regions could either be due to linkage drag of genes located in the polymorphic regions in isogenic line contrasts without effect on SCMV resistance (caused by heterozygosity), or clustering of genes involved in SCMV resistance in the *Scmv*1 and/or *Scmv2 *regions. Assuming that each of the two donor segments is 40 cM long, both regions would represent 5% of the total genome (80 cM out of 1600 cM average maize genome size). The percentage of candidate genes (one gene each) falling into either the *Scmv1 *(6.00–6.01) or *Scmv2 *bins (3.04–3.05) is 8% (2 out of 24 putative map locations for the 14 mapped differentially expressed genes, Table 2), which is not significantly different from the 0-hypothesis tested by the X^2 ^test (no clustering of differentially expressed genes). Thus, no evidence of clustering of differentially expressed genes in the *Scmv1 *and *Scmv2 *regions was found, which also means, that differential gene expression of genes due to linkage drag was limited. Moreover, finding 2 out of 24 gene locations in agreement with the *Scmv1 *and *Scmv2 *genome locations is not significantly different from expectations based on probability theory. Thus, colocalization of differentially expressed genes is only a weak indicator for candidacy of being *Scmv1 *or *Scmv2*.

#### Time course data

In contrast to fungi and bacteria, viruses are directly transferred by specific vectors into host cells. The infection cycle includes virus disassembly, RNA translation and replication, new viral particle assembly, and movement. The time required for these processes may vary and be virus/host dependent.

Immediate response of plants against virus attack is obligatory for fast activation of defence mechanisms [32]. Significant differential expression of majority of genes in our study one hour post inoculation, dropping down to about one-third twelve hours post inoculation may be a result of such rapid responses of host plant to viral infection. The anticipation of detected genes in mechanical stress responses proved insignificant in applied mock control experiment.

To arque our statement we present the potyvirus study of Maule et al. [33], where induction of genes related to pathogenesis (putative protein targeting, virion assembly and trafficking) and to general stress responses was detected immediately after inoculation, similar to the study of Love et al. [34]. Furthermore, Marathe et al. [35], detected robust plant resistance responses at transcriptome level to potyvirus infection at early (at least three hours post inoculation) time points. Changes in gene expression due to responses initiated by specific interactions between virus and host proteins, for instance potyvirus coat protein VPg with plant eIF4 initiation factor have also been reported [36]. Moreover, rapid silencing and blockade of viral protein expression by modified RNAi was observed within one-two hours post inoculation [37]. In contrast, however Yang et al. [38] concluded that changes in gene expression depend on virus type and its accumulation (threshold of viral RNA and proteins) in infected tissues hence occur rather at later stages post inoculation. Similarly, Whitham et al. [39] stated that actual transcriptional changes depend on the progress of viral infection.

The conflicting observations of different research groups might be due to host-virus system specificity and need to be studied in more detail, before generalizations can be made.

#### Candidate genes and their involvement in signal transduction pathways

Metallothioneins are known to be involved in metal binding/metabolism and detoxification reactions in animals and yeasts [40,41]. Slightly modified functions of metallothioneins have been reported in plants, where their increased expression was observed in senesced leaflet and abscission zones, under ethylene induction, or as a consequence of mechanical wounding when infecting tobacco with TMV virus was obsereved [42-44]. Moreover, a possible role of metallothioneins in controlling intracellular redox potential and activation of oxygen detoxification, a common strategy used by plants after pathogen invasion was suggested by Hamer [40]. Finding of metallothionein-like protein expressed in all time points (including mock control at significant fold of change) and for both viruses may suggest its differential expression as a cause of mechanical wounding. However, its participation in pathogen control cannot be ruled out.

S-adenosylmethionine synthetase is a key enzyme involved in generation of S-adenosylmethionine from methionine. S-adenosylmethionine is a major methyl donor in plants involved in polyamin and ethylene biosynthesis as well as in methylation reactions modifying lipids, proteins and nucleic acids [45-47]. Ethylene plays an important role in various plant disease resistance pathways. It has originally been considered a stress hormone due to its synthesis induced by stress signals, such as mechanical wounding, chemicals and metals, drought, extreme temperatures, and pathogen infection [46,48]. Some pathogens can induce plant defence responses via activation of the ethylene signal transduction pathway, whereas plants deficient in ethylene signalling may show either increased susceptibility or increased resistance [49,50]. Alternatively, methylation of the fully susceptible F7 ^SS/SS ^genotype might be reduced as revealed in our study by the upregulation of S-adenosylmethonine synthetase isoforms (*946126A02.y1*, *1091032B12.y1 a *and *b*, *946063C12.y1*) in all other genotypes. Resistance to SCMV and MDMV might depend on the methylation status of the plant, relating to post-transcriptional gene silencing mediated by HEN1 like methyltransferase [37].

Germins are water-soluble proteins expressed during seed germination in very young seedlings of wheat and barley. In mature leaves they are induced in response to pathogen attack [51]. In plants other than wheat and barley, sequences related to germins are termed "germin-like". Germins and germin-like proteins were isolated from hot pepper during resistance response to bacterial and viral infection [52,53]. Pathogen response functions of germins were discovered with the identification of germin as an oxalate oxidase generating hydrogen peroxide. H_2_O_2 _is a catalyst of cell-wall reinforcement (oxidative cross-linking) and a basis for defence reactions in higher plants. The specific-pathogen-response OXO transcript was found in the wall of barley mesophyll cells six hours after inoculation with powdery mildew [54]. However, it is still unclear if germin-like proteins also have oxalate-oxidase activity and if their biological function is comparable to germins [55-59].

A common feature of expression patterns of genes in the resistant genotype F7 ^RR/RR ^is lack of signs of oxidative damage (downregulation of class III peroxidases and germins), whereas partially resistant and susceptible genotypes showed upregulation of genes controlling production of hydrogen peroxide. Furthermore, oxidative damage could affect chloroplasts, perturbing their proper function as shown for response to plum pox potyvirus [60]. The analysis of SCMV cylindrical inclusion (CI) virus protein (NP_734137), known to be involved in virus replication and cell-to-cell movement [61], with the ChloroP 1_1 CBS tool  revealed a possible chloroplast transit peptide of 65 amino acids [62]. This could explain upregulation of the 14-3-3 proteins (*Zm06_09h07_R*) in genotypes other than the fully resistant genotype F7 ^RR/RR^. 14-3-3 proteins are known to target the transit peptide to the chloroplast, where it will be cleaved upon entrance as shown for other potyvirus [63].

The putative antiviral function of 26S ribosomal RNA gene in ribosome depurination and blocking of translation of viral genetic materials was reported by Taylor et al. [64]. Other genes putatively related to pathogenesis, coding for example a calcium dependent protein kinase, cytochrome c, a zinc finger protein, a peroxidase precursor, an ubiquitin-conjugating enzyme, 40S ribosomal protein or eukaryotic translation initiation factor 4E, were identified in our microarray assay. The involvement of calcium dependent protein kinase (CDPK) in defence signalling has been investigated in transgenic tobacco cell cultures, where CDPK was found to be activated in Avr9/Cf-9 gene-for-gene-dependent signal transduction, as well as in tobacco leaves after Avr9/Cf-9 interaction and hypoosmotic stress [65,66]. Its participation in signal transduction pathways in *Arabidopsis *infected with cucumber mosaic virus was demonstrated by Marathe et al. [35]. Cytochrome c has been previously reported to give an apoptotic-like response under *Agrobacterium *infection of maize suspension cells and was called a cell death inducer released from mitochondria during ROS-induced programmed cell death in plants [67-69]. Furthermore, zinc finger proteins were found to be induced by various types of stresses (for example ethylene treatment), under viral and fungal inoculation [38,70]. An *Arabidopsis *zinc-finger protein encoded by the *LSD1 *gene acts as a negative regulator of hypersensitive response to restrict the spreading of cell death [71]. Class III peroxidases (plant-specific oxidoreductase) participate in lignification, suberization, wound healing and defence against pathogen infection. Increased mRNA levels of POX and its increased activity was previously reported in plants upon mechanical wounding in various plants [72,73], [74-77]. Finally, altered expression of ubiquitin-conjugating enzyme, 40S ribosomal protein, and eukaryotic translation initiation factor 4E were identified under infection with various potyviruses [33,38,35,78].

## Conclusion

In summary, based on the results of our custom microarray, the majority of differentially expressed genes belong to the oxidative and methylation pathways, as well as pathways involved in primary and secondary responses to virus attack. Oxidative insensitivity and methylation status of the F7 ^RR/RR ^genotype seem to play important roles for resistance to SCMV and MDMV, pointing towards post-transcriptional gene silencing as major underlying defence mechanism.

The presented data indicate successful identification of similar expression patterns between mono- and dicotyledonous species and deliver new insights into the defense response mechanisms of monocot plants against potyviruses. In future, application of complementary technologies, such as transgenic approaches, infection studies with potyviruses including green fluorescent protein, virus-induced gene silencing or global proteome profiling will allow further and in-depth verification of the data.

## Methods

### Plant material

Four near-isogenic homozygous maize (*Zea mays *L.) genotypes were produced at Research Centre Flakkebjerg, Denmark. The SCMV resistant near-isogenic line F7 ^RR/RR ^(with introgressions at two genome regions conferring resistance to SCMV on chromosomes 3 and 6) was derived from a cross between Flint line F7 (susceptible to SCMV) and Dent line FAP1360A (completely resistant to SCMV) after seven backcrosses to F7 and three selfing generations [20]. F7 ^RR/SS ^(resistance allele from FAP1360A fixed at *Scmv2*) and F7 ^SS/RR ^(resistance allele from FAP1360A fixed at *Scmv1*) were derived from F7 ^RR/RR ^by applying SSR markers in the *Scmv1 *and *Scmv2 *regions after an initial cross and subsequent selfings.

### Design of greenhouse trials

Plants were grown under controlled greenhouse conditions for 14 days before virus inoculation at ~24°C during the day and ~18°C at night. Plants inoculated with SCMV or MDMV were grown in separate greenhouse cabins in order to avoid cross-contamination. SCMV infected plants were grown in five blocks (= time points) with four biological replicates each, four near-isogenic genotypes within replicates and eight plants per genotype and replicate. Each plant was grown in a separate pot and eight plants of the same genotype were arranged in rows within biological replications. A sixth block was used for mock control.

The MDMV infected plants were grown in two blocks (= time points), with four biological replicates each, two near-isogenic genotypes per replicate (F7 ^RR/RR ^and F7 ^SS/SS^), and eight plants per genotype and replicate in separate pots, like in the SCMV experiment.

### Inoculation and harvest of leaf material

14 days after sowing leaf samples for the SCMV experiment were harvested at time point one (T1: before inoculation), followed by mechanical rub inoculation and harvesting at time point 2 (T2: one hour after inoculation), time point 3 (T3: six hours after inoculation), time point 4 (T4: twelve hours after inoculation) and time point 5 (T5: twenty four hours after inoculation). Mock control plants were inoculated with water and harvested before inoculation with SCMV (one our after "inoculation"). Mock plants were assigned as T9. Plants for the MDMV experiment were harvested at time points T2 and T3, while T1 and T9 samples were utilised from the SCMV experiment. The four youngest and fully developed leaves from eight plants per genotype and replication were inoculated, subsequently harvested, quick-frozen and stored at -80°C.

The SCMV inoculation mixture was prepared from 4–5 young leaves of SCMV inoculated susceptible F7 ^SS/SS ^adult plants displaying typical mosaic symptoms, homogenized in five volumes of a 0.01 M phosphate buffer (pH 7.0) and mixed with carborundum. SCMV isolate "Seehausen" was utilized. MDMV inoculation was performed in the same way using a highly pathogenic Italian MDMV isolate.

To evaluate the infection ratio for both viruses, inoculated susceptible F7 ^SS/SS ^plants were grown for additional two weeks after leaf harvest for RNA experiments to determine mosaic symptoms. Those plants, where leaves were harvested one hour after inoculation, were validated for the presence of virus symptoms after two weeks.

### Sample preparation

mRNA was isolated from sixteen randomly chosen leaves (half of the harvested stock) per entry and replication using DynaBeads oligo(dT)_25 _(Dynal Biotech, Oslo, Norway). Reverse transcription was performed with SuperScript II (Invitrogen GmbH, Karlsruhe, Germany) and second strand synthesis by Klenow DNA polymerase I (Fermentas Life Sciences, St. Leon-Rot, Germany) on Dynabeads with incorporation of aa-dUTP's. Samples were labelled with Cy3 and Cy5 (Amersham Pharmacia, Piscataway, NJ, USA) and unincorporated dyes were purified with QiaQuick PCR purification kit (QiaGen, Hilden, Germany) according to manufacturer's recommendations. The amount of labelled product was measured spectrophotometrically in a 50 μl quartz cuvette (Cy3-550 nm, Cy5-650 nm). 30 to 60 pmol of Cy3/Cy5 labeled cDNAs were applied to the microarray (Gregersen et al., 2005). Arrays were scanned using GeneTac UC 4 × 4 microarray scanner (GeneMachines™, Genomic Solutions Inc, USA). Quantification was done using Array Vision software (version 8.0, Imaging Research Inc., St. Catharines, Ontario, Canada). The spot grids were manually aligned with the spots for each slide. Details on experimental data are available through EMBL-EBI ArrayExpress  with the accession number E-TABM-586.

### SCMV cDNA array fabrication

Maize genes pre-selected for SCMV resistance in preceding experiments [18,19] cloned into ten different *E. coli *vectors were obtained from the Arizona BAC/EST resource centre and from the Schnable Lab, Iowa State University, USA, as stab cultures. Resistance genes and resistance gene analogues were obtained from China Agricultural University, Beijing. Plasmid mini-preps were conducted using R.E.A.L^® ^Prep 96 Kit (QiaGen AG, Hilden, Germany) according to manufacturer's instructions and PCR amplification was done using primers designed for each vector (Primer Express™ software, version 1.5, Applied Biosystems, Foster City, USA) (see Additional file 6). Quality of PCR products was checked on 1.5% agarose gels and quantified by GelPro Analyzer software version 3.1 (Media Cybernetics, Inc., Silver Spring, USA). Samples were purified and desalted using ethanol/acetate precipitation (130 μl of EtOH/acetate mix per 50 μl of PCR products). Subsequently, pellets were dissolved in variable amounts of 50% dimethyl sulphoxide (DMSO) to the final concentration of 420 ng/μl and 5 μl of each sample was transferred into 384 well plates and spotted to Nexterion Slides A+ (SCHOTT JENA^er ^GLAS GmbH, Jena, Germany) using the Qarray mini microarray spotter with 16 pins (Genetix GmbH, Munich, Germany). Samples were spotted in triplicate in a 9 × 9 pin group design with 16 pin groups on the chip. After spotting, arrays were air-dried and DNA was cross-linked to the slides by UV irradiation at 450 mJ (Stratalinker, Stratagene). Before hybridization slides were baked at 80°C for 45 – 60 min, boiled in 1 × SSC for 3–5 min to remove access DNA, blocked according to Nexterion blocking protocols, and stored in an exsiccator in dark containers until usage.

The "SCMV array" contained 878 spots tri-plicated (technical replications) across the slide, including 110 wheat controls, 6 maize controls (2 single and 2 doubled), 302 resistance genes and resistance gene analogues (RGAs) from the China Agricultural University (CAU), Beijing (Prof. Mingliang Xu), 451 differentially expressed genes identified in a previous SCMV study [18,19], 3 published RGAs: pic 13 and pic 19 with duplication [79], and 3 exons from the eIF3E barley gene with duplication.

### Hybridization design

144 and 24 arrays were utilised for the SCMV and the MDMV experiment, respectively. The SCMV experiment was carried out with all four near-isogenic genotypes. An unresolvable row-column design was optimized for six possible pairings of genotypes within each time point, where six rows corresponded to six slides and two columns corresponded to the two dyes. The MDMV experiment was carried out with two near isogenic genotypes: F7 ^SS/SS ^and F7 ^RR/RR^, using a pair-wise dye-swap design.

### Quantitative RT-PCR

Total RNA from leaf tissue (remaining sixteen harvested leaves per biological replication) of near isogenic genotypes was isolated using TRIzol reagent (Invitrogen GmbH, Karlsruhe, Germany). RNA purification was conducted on RNeasy mini kit columns (QiaGen AG, Hilden, Germany) following manufacturer's instructions, with previous DNA digestion with RNase free DNase (Qiagen AG, Hilden, Germany). RNA quality was checked on 1.2% formaldehyde agarose gels and quantification was done by spectrophotometry. Sequence-specific primers for real-time (RT) PCR were designed using Primer Express™ software, Version 1.5 (Applied Biosystems, Foster City, CA, USA) (Table 5).

**Table 5:** Sequence specific primers for reference and target genes for qRT-PCR SCMV experiment

**Gene name**	**Primer sequence (5' - 3')**	**Annealing temp.**
*Maize actin 1*	For : TCC TGA CAC TGA AGT ACC CGA TTG Rev: CGT TGT AGA AGG TGT GAT GCC AGT T	56.0°C/60.5°C
*26S ribosomal RNA gene*	For : CAT TCA ATC GGT AGG AGC GAC Rev: GGT CTT CAA CGA GGA ATG CC	60.5°C
*Metallothionein-like protein*	For : ACT CGG CCC ACA CAG CA Rev: GAG ATG TTG GCG CCG TG	60.5°C
*S-adenosylmethionine synthetase 1*	For : CCT ATC GGT GTT CGT GGA CA Rev : TGA TCA TGC CGG GCC T	60.5°C
*14-3-3-like protein GF14-6*	For : GGG AGC CCC CAA ATT TTA CT Rev: AGT GTT TGC TGC TGT CGA ATG	60.5°C
*S-adenosylmethionine synthetase 1*	For : TCC CAA AAC TGA GCT TGA AGC Rev: GCA GTC TTT GGA TCA AAG CCA	56.0°C
*Germin-like protein 4*	For : CCC GTC GAA GAA GAA GTC GT Rev: CTT GCT GCT GAC CCC GTA C	56.0°C

QRT-PCR was conducted with One-Step QuantiTect SYBR^® ^Green RT-PCR Kit (Qiagen AG, Hilden, Germany) on the ABI PRISM™ 7700 Sequence Detection System (Applied Biosystems, Foster City, CA, USA) under the following conditions: 50°C for 30 min, 95°C for 15 min and 45 cycles of 94°C for 30 sec, 58°C for 15 sec, and 72°C for 30 sec in total volumes of 25 μl reactions. Four biological and three technical replications were used for every gene in order to precisely quantify transcript abundance. Dissociation curve analyses were performed to identify primer-dimers and unspecific PCR products. An endogenous reference sequence was derived from the maize actin 1 gene (MAc1) [EMBL-EBI: J01238]

### Statistics

#### Microarrays

Raw intensity and background values generated by Array Vision, version 8.0 (Imaging Research Inc., St. Catharines, Canada) were utilized for data analysis. The main interest was to determine the expression patterns of pair-wise contrasts between genotypes at the same time point (within-time-point analysis), whereas contrasts of a genotype at two different time points were of secondary interest (between-time-point analysis). Locally weighted scatterplot smoothing (LOWESS) regression was performed to adjust for differences within an array.

The following linear mixed model was fitted:

*y*_*ijkl *_= *g*_*i *_+ *t*_*i *_+ *a*_*k *_+ *d*_*l *_+ (g*t)_*ij *_+ (g*d)_*il *_+ *e*_*ijkl*_,

where *y*_*ijkl *_is the log2-signal intensity, *g*_*i *_fixed effect for genotype, *t*_*j *_fixed effect for the time point, *a*_*k *_random effect for the array, *d*_*l *_fixed effect for the dye, (g*t)_*ij *_genotype and time point interaction and (g*d)_*il *_genotype and dye interaction. The calculations were performed with the SAS System for Windows, Version 9.1.

Pair-wise contrasts between different genotype*time combinations in the SCMV experiment were estimated, considering only contrasts between genotypes within one time point and contrasts of one genotype at different time points. The corresponding FDR adjusted p-values and fold changes were determined. Least square means of genotype*time were calculated, i.e., the value of a certain genotype at a specific time point averaged over the other effects. The degrees of freedom for the tests were calculated according to the containment method. SAS (Institute Inc. (1999): SAS/STAT User's Guide, Version 8. Cary, NC). For MDMV data analysis the same linear model was fitted but separate variance terms for mock control and normal data were specified.

Blastn analysis in TIGR Unique Gene Indices  for maize was performed in order to reveal the putative function of unknown sequences from *Arabidopsis thaliana*, barley, maize, rice, rye and wheat, with a cut off e-value of 10 (Ros et al., 2004). Additional blastn analyses were performed in MIPS  and IRGSP  databases for gaining maximum information about the genes of interest.

The calculations of significances for the number of genes between time points were calculated using the McNemar exact test (SAS System for Windows, Version 9.1).

#### QRT-PCR

Relative expression rates of the target genes were calculated as follows:

$$rel.expression=\frac{{\left(1+Etarget\right)}^{\Delta Cttarget}}{{\left(1+Eref\right)}^{\Delta Ctref}},$$

where *E*_*target *_is the PCR efficiency for the target gene and *E*_*ref *_is the PCR efficiency for the endogenous reference. PCR efficiencies (*E = 10*^*(-1/slope)*^-*1*), were derived from calibration data of serially diluted RNA: 100%, 50%, 10%, 5%, 1%, 0.5%, 0.1% and water. ΔCt_target _and ΔCt_ref _values were determined as described by Dilger et al. [80]. Baseline and threshold values were adjusted manually if necessary, as recommended by Applied Biosystems .

## Abbreviations

cM: centimorgan; Cy3: cyanine 3; Cy5: cyanine 5; EST: Expressed Sequence Tag; FDR: false discovery rate; GO: gene ontology; H_2_O_2_: hydrogen peroxide; IRGSP: International Rice Genome Sequencing Project; JGMV: Johnsongrass Mosaic Virus; MIPS: Munich Information Center for Protein Sequences; MDMV: Maize Dwarf Mosaic Virus; NIL: near-isogenic line; PCR: polymerase chain reaction; qRT-PCR: quantitative real-time polymerase chain reaction; QTL: quantitative trait locus; RGA: resistance gene analogue; RNA: ribonucleic acid; ROS: reactive oxygen species; SCMV: Sugarcane Mosaic Virus; SSH: Suppression Subtractive Hybridization; SrMV: Sorghum Mosaic Virus; T1-T9: time point 1–9; TIGR: The Institute for Genomic Research; X^2^: Chi-square test.

## Authors' contributions

AU prepared cDNAs for spotting SCMV arrays, conducted inoculation and harvesting of maize plants with SCMV and MDMV viruses and carried out all microarray experiments, GD designed the SCMV array layout, performed cDNAs quantification for spotting, fabricated SCMV arrays and submitted microarray data into ArrayExpress, BS undertook bioinformatic analysis of microarray data and was involved in outcome discussion, H-PP prepared statistical design for microarray experiments, coordinated data analysis and participated in the discussion of results, MX donated clones of resistant genes and resistant gene analogues, CRI coordinated and supervised the greenhouse design and plant inoculations with both viruses, GW and TL were project initiators and supervisors, participated in the discussion of all experimental parts of the project and preparation of the manuscript.

All authors read and approved the final manuscript.

## Supplementary Material

Additional file 1 **SCMV within-time-point significantly differentially expressed sequences.** File 1 illustrates the 65 significantly differentially expressed sequences identified within time points in the SCMV experiment. All information available for the genes is provided in the file. Click here for file

Additional file 2 **SCMV between-time-point significantly differentially expressed sequences.** File 2 illustrates the 28 significantly differentially expressed sequences identified between time points in the SCMV experiment, and gives basic information about the genes retrieved from the analysis. Click here for file

Additional file 3 **Genes up-regulated in single MDMV time points.** File 3 illustrates the percentages of all differentially expressed vs. significantly differentially expressed up-regulated genes identified in the between-time-point MDMV experiment for each of the four applied time points (including mock control). Click here for file

Additional file 4 **Genes expressed in the MDMV experiment based on their folds of change.** File 4 illustrates the percentages of all differentially expressed vs. significantly differentially expressed genes in the between-time-point MDMV experiment distributed according to their folds of change. Click here for file

Additional file 5 **MDMV-between-time-point significantly differentially expressed sequences.** File 5 illustrates the 2 significantly differentially expressed sequences identified within time points in the MDMV experiment, and gives basic information about the genes retrieved from the analysis. Click here for file

Additional file 6 **Vectors and primers for insert amplification.** File 6 illustrates the 10 different *E. coli *vectors and their primer sequences utilised in this study for the amplification of inserts to be spotted on the SCMV cDNA microarray. Click here for file
