# Supplementary material for: Validation of candidate genes putatively associated with resistance to SCMV and MDMV in maize (Zea mays L.) by expression profiling
Source: BMC Plant Biol. 2009 Feb 2;9:15. doi: 10.1186/1471-2229-9-15 (PMC2669481; doi:10.1186/1471-2229-9-15)
Supplement: Additional file 1 — SCMV within-time-point significantly differentially expressed sequences. File 1 illustrates the 65 significantly differentially expressed sequences identified within time points in the SCMV experiment. All information available for the genes is provided in the file. [file 1471-2229-9-15-S1.doc]

| **EST** | **GA#** | **Time point** | **Genotype 1** | **Genotype 2** | **TIGR homology: blast n** | **MIPS homology: blast n** | **IRGSP homology: blast n** | **Fold of change** | **Up-regulated** | **FDR p ≤ 0.05** |
| --- | --- | --- | --- | --- | --- | --- | --- | --- | --- | --- |
| *605018B03.x1* | AI668198 | 2 | F7 ^RR/RR^ | F7 ^SS/RR^ | gb|AF036494.1|AF036494 Eucryphia lucida large subunit 26S ribosomal RNA gene, partial sequence, partial (52%) | - | expressed protein | 2.07 | F7 ^SS/RR^ | 2.32E-07 |
| *605018B03.x1* |  | 2 | F7 ^RR/RR^ | F7  ^RR/SS^ |  |  |  | 1.95 | F7 ^RR/SS^ | 1.66E-06 |
| *605018B03.x1* |  | 3 | F7 ^SS/SS^ | F7 ^RR/RR^ |  |  |  | 1.79 | F7 ^SS/SS^ | 0.000152 |
| *605018B03.x1* |  | 2 | F7 ^SS/SS^ | F7 ^RR/RR^ |  |  |  | 1.7 | F7 ^SS/SS^ | 0.000206 |
| *605018B03.x1* |  | 9 | F7 ^SS/SS^ | F7 ^RR/RR^ |  |  |  | 1.64 | F7 ^SS/SS^ | 0.004848 |
| *605018B03.x1* |  | 9 | F7 ^RR/RR^ | F7 ^SS/RR^ |  |  |  | 1.60 | F7 ^SS/RR^ | 0.007555 |
| *605018B03.x1* |  | 3 | F7 ^RR/RR^ | F7 ^SS/RR^ |  |  |  | 1.51 | F7 ^SS/RR^ | 0.047531 |
| *605018B04.x1* | AI668199 | 2 | F7 ^RR/RR^ | F7 ^SS/RR^ | UP|Q5U7K6_9POAL (Q5U7K6) Metallothionein-like protein, partial (94%) | metallothionein-like protein (AtMT-K) | metallothionein-like protein, type 2, putative, expressed | 2.61 | F7 ^SS/RR^ | 0 |
| *605018B04.x1* |  | 9 | F7 ^SS/SS^ | F7 ^RR/RR^ |  |  |  | 2.54 | F7 ^SS/SS^ | 0 |
| *605018B04.x1* |  | 9 | F7 ^RR/RR^ | F7 ^SS/RR^ |  |  |  | 2.97 | F7 ^SS/RR^ | 0 |
| *605018B04.x1* |  | 2 | F7 ^SS/SS^ | F7 ^RR/RR^ |  |  |  | 2.42 | F7 ^SS/SS^ | 2.44E-12 |
| *605018B04.x1* |  | 3 | F7 ^SS/SS^ | F7 ^RR/RR^ |  |  |  | 2.41 | F7 ^SS/SS^ | 2.44E-12 |
| *605018B04.x1* |  | 3 | F7 ^RR/RR^ | F7 ^SS/RR^ |  |  |  | 2.19 | F7 ^SS/RR^ | 8.4E-10 |
| *605018B04.x1* |  | 9 | F7 ^RR/RR^ | F7  ^RR/SS^ |  |  |  | 2.05 | F7 ^RR/SS^ | 1.2E-07 |
| *605018B04.x1* |  | 2 | F7 ^RR/RR^ | F7  ^RR/SS^ |  |  |  | 1.96 | F7 ^RR/SS^ | 1.66E-06 |
| *605018B04.x1* |  | 4 | F7 ^RR/RR^ | F7 ^SS/SS^ |  |  |  | 1.86 | F7 ^SS/RR^ | 1.08E-05 |
| *605018B04.x1* |  | 5 | F7 ^SS/SS^ | F7 ^RR/RR^ |  |  |  | 1.64 | F7 ^SS/SS^ | 0.003079 |
| *605018B04.x1* |  | 3 | F7 ^SS/SS^ | F7  ^RR/SS^ |  |  |  | 1.61 | F7 ^SS/SS^ | 0.005143 |
| *605018B04.x1* |  | 5 | F7 ^RR/RR^ | F7 ^SS/RR^ |  |  |  | 1.58 | F7 ^SS/RR^ | 0.010133 |
| *606007B06.x1* | AI691713 | 3 | F7 ^SS/SS^ | F7 ^RR/RR^ | UP|Q6ZFT9_ORYSA (Q6ZFT9) Putative diphosphate-fructose-6-phosphate 1-phosphotransferase alpha chain | - | pyrophosphate-fructose 6-phosphate 1-phosphotransferase alpha subunit, putative, expressed | 1.51 | F7 ^SS/SS^ | 2.62E-05 |
| *606007B06.x1* |  | 3 | F7 ^SS/SS^ | F7  ^RR/SS^ |  |  |  | 1.34 | F7 ^SS/SS^ | 0.027941 |
| *606021F11.x2* | AI714860 | 5 | F7 ^SS/SS^ | F7  ^RR/SS^ | UP|Q5ZBB1_ORYSA (Q5ZBB1) Dual-specific kinase DSK1-like, partial (13%) | protein kinase, putative | ATP binding protein, putative, expressed | 2.33 | F7 ^SS/SS^ | 0.002316 |
| *614013G06.x1* | AI861424 | 3 | F7 ^SS/SS^ | F7 ^SS/RR^ | homologue to UP|CYC_MAIZE (P00056) Cytochrome c, complete | cytochrome C like protein | cytochrome c, putative, expressed | 1.64 | F7 ^SS/SS^ | 0.040567 |
| *614044F12.x4* | AI978054 | 1 | F7 ^SS/SS^ | F7  ^RR/SS^ | UP|Q96295_ARATH (Q96295) Calcium-dependent protein kinase (Fragment), partial (61%) | calcium-dependent like protein kinase | calcium-dependent protein kinase, isoform AK1, putative, expressed | 1.42 | F7 ^RR/SS^ | 0.00017 |
| *614044F12.x4* |  | 1 | F7 ^SS/SS^ | F7 ^RR/RR^ |  |  |  | 1.36 | F7 ^RR/RR^ | 0.002812 |
| *614044F12.x4* |  | 4 | F7 ^SS/RR^ | F7  ^RR/SS^ |  |  |  | 1.33 | F7 ^SS/RR^ | 0.010551 |
| *614044F12.x4* |  | 4 | F7 ^RR/RR^ | F7 ^SS/RR^ |  |  |  | 1.31 | F7 ^SS/RR^ | 0.022349 |
| *945031C10.x1* | BE129848 | 2 | F7 ^RR/RR^ | F7 ^SS/RR^ | similar to UP|Q44086_ACILW (Q44086) Orf1 and esterase (est) genes, . precursor, partial (11%) | KED - like protein | expressed protein | 7.60 | F7 ^RR/RR^ | 0.034466 |
| *949062B09.y1* | BI430868 | 2 | F7 ^RR/RR^ | F7  ^RR/SS^ | UP|RBS_MAIZE (P05348) Ribulose bisphosphate carboxylase small chain, chloroplast precursor (RuBisCO small subunit) , complete | ribulose bisphosphate carboxylase like protein, small subunit | ribulose bisphosphate carboxylase small chain C, chloroplast precursor, putative, expressed | 1.54 | F7 ^RR/SS^ | 0.013406 |
| *MEST12-E11.T3* | BG840850 | 4 | F7 ^RR/RR^ | F7 ^SS/RR^ | RF|XP_507584.1|51979675|XM_507584 P0413H11.35 gene product {Oryza sativa (japonica cultivar-group)} (exp=-1; wgp=0; cg=0), partial (94%) | light-harvesting chlorophyll a/b-binding protein (Cab4) | chlorophyll a-b binding protein 4, chloroplast precursor, putative, expressed | 1.31 | F7 ^SS/RR^ | 0.000346 |
| *MEST12-E11.T3* |  | 4 | F7 ^SS/RR^ | F7  ^RR/SS^ |  |  |  | 1.28 | F7 ^SS/RR^ | 0.001601 |
| *MEST12-E11.T3* |  | 4 | F7 ^SS/SS^ | F7 ^SS/RR^ |  |  |  | 1.28 | F7 ^SS/RR^ | 0.002328 |
| *MEST12-E11.T3* |  | 2 | F7 ^RR/RR^ | F7 ^SS/RR^ |  |  |  | 1.26 | F7 ^SS/RR^ | 0.005974 |
| *MEST19-G10.T3* | BG841285 | 2 | F7 ^SS/SS^ | F7 ^RR/RR^ | UP|Q6H5U3_ORYSA (Q6H5U3) Putative sphingolipid delta 4 desaturase | dehydrin Xero2 | abscisic stress ripening protein 1, putative, expressed | 1.26 | F7 ^SS/SS^ | 0.031991 |
| *MEST22-A03.T3* | BG841470 | 9 | F7 ^SS/RR^ | F7  ^RR/SS^ | UP|P93641_MAIZE (P93641) S-like RNase, complete | - | extracellular ribonuclease LE precursor, putative, expressed | 1.39 | F7 ^RR/SS^ | 0.021042 |
| *MEST24-E10.T3* | BG841717 | 2 | F7 ^RR/RR^ | F7 ^SS/RR^ | UP|Q3BCU2_MAIZE (Q3BCU2) Zinc finger protein, complete | unknown protein | zinc finger A20 and AN1 domains-containing protein, putative, expressed | 1.33 | F7 ^SS/RR^ | 0.000854 |
| *MEST24-E10.T3* |  | 2 | F7 ^SS/SS^ | F7 ^RR/RR^ |  |  |  | 1.25 | F7 ^SS/SS^ | 0.034013 |
| *MEST24-G11.T3* | BG841741 | 2 | F7 ^RR/RR^ | F7 ^SS/RR^ | RF|XP_506910.1|51964250|XM_506910 OSJNBa0057G07.4 gene product {Oryza sativa (japonica cultivar-group)} (exp=-1; wgp=0; cg=0) | PSI-L protein (PsaL, PSI subunit XI) | photosystem I reaction center subunit XI, chloroplast precursor, putative, expressed | 1.28 | F7 ^SS/RR^ | 0.002383 |
| *MEST24-G11.T3* |  | 2 | F7 ^SS/SS^ | F7 ^RR/RR^ |  |  |  | 1.22 | F7 ^SS/SS^ | 0.05026 |
| *MEST40-B08.T3* | BG842796 | 2 | F7 ^SS/SS^ | F7 ^RR/RR^ | unknown protein {Oryza sativa (japonica cultivar-group)} (exp=0; wgp=1; cg=0), partial (91%) | unknown protein | expressed protein | 1.34 | F7 ^SS/SS^ | 0.004307 |
| *MEST40-B08.T3* |  | 5 | F7 ^RR/RR^ | F7  ^RR/SS^ |  |  |  | 1.31 | F7 ^RR/SS^ | 0.019342 |
| *MEST40-B08.T3* |  | 2 | F7 ^RR/RR^ | F7  ^RR/SS^ |  |  |  | 1.28 | F7 ^RR/SS^ | 0.042609 |
| *MEST40-G05.T3* | BG842844 | 2 | F7 ^RR/RR^ | F7 ^SS/RR^ | UP|Q8S920_ORYSA (Q8S920) Ubiquitin-conjugating enzyme OsUBC5a, complete | ubiquitin-protein ligase UBC9 | ubiquitin-conjugating enzyme E2-17 kDa 9, putative, expressed | 1.28 | F7 ^SS/RR^ | 0.042609 |
| *MEST41-B03.T3* | BG842878 | 4 | F7 ^RR/RR^ | F7 ^SS/RR^ | UP|Q9FUL7_MAIZE (Q9FUL7) 40S ribosomal protein S24, complete | putative ribosomal protein s19 or s24 | 40S ribosomal protein S24, putative, expressed | 1.43 | F7 ^SS/RR^ | 0.031991 |
| *MEST63-E12.T3* | BM073273 | 2 | F7 ^RR/RR^ | F7 ^SS/RR^ | UP|Q5N7Q9_ORYSA (Q5N7Q9) Phosphatidic acid phosphatase beta-like, partial (77%) | putative phosphatidic acid phosphatase | lipid phosphate phosphatase 3, chloroplast precursor, putative, expressed | 1.35 | F7 ^RR/RR^ | 0.020598 |
| *MEST67-A07.T3* | BM073434 | 4 | F7 ^SS/RR^ | F7  ^RR/SS^ | UP|Q2XX96_ZEAMP (Q2XX96) Pathogenesis-related protein 5, complete | thaumatin-like protein | thaumatin-like protein, precursor, putative, expressed | 1.34 | F7 ^RR/SS^ | 0.000214 |
| *MEST67-A07.T3* |  | 4 | F7 ^RR/RR^ | F7  ^RR/SS^ |  |  |  | 1.27 | F7 ^RR/SS^ | 0.01323 |
| *MEST67-A07.T3* |  | 3 | F7 ^RR/RR^ | F7  ^RR/SS^ |  |  |  | 1.24 | F7 ^RR/SS^ | 0.042609 |
| *MEST82-F04.T3* | BM074185 | 2 | F7 ^RR/RR^ | F7  ^RR/SS^ | - | putative protein | - | 1.31 | F7 ^RR/RR^ | 0.005148 |
| *MEST82-F04.T3* |  | 2 | F7 ^RR/RR^ | F7 ^SS/RR^ |  |  |  | 1.31 | F7 ^RR/RR^ | 0.005188 |
| *MEST82-F04.T3* |  | 2 | F7 ^SS/SS^ | F7 ^RR/RR^ |  |  |  | 1.27 | F7 ^RR/RR^ | 0.034585 |
| *MEST333-H11.T3* | BM341362 | 2 | F7 ^RR/RR^ | F7 ^SS/RR^ | UP|IM30_ORYSA (Q8S0J7) Probable membrane-associated 30 kDa protein, chloroplast precursor, complete | unknown protein | membrane-associated 30 kDa, protein, chloroplast precursor, putative, expressed | 1.56 | F7 ^SS/RR^ | 0.008593 |
| *Zm06_09h07_R* | BG836057 | 2 | F7 ^RR/RR^ | F7 ^SS/RR^ | UP|14331_MAIZE (P49106) 14-3-3-like protein GF14-6, complete | - | 14-3-3-like protein GF14-6, putative, expressed | 1.62 | F7 ^SS/RR^ | 1.08E-05 |
| *Zm06_09h07_R* |  | 2 | F7 ^RR/RR^ | F7  ^RR/SS^ |  |  |  | 1.57 | F7 ^RR/SS^ | 9.45E-05 |
| *Zm06_09h07_R* |  | 4 | F7 ^SS/SS^ | F7 ^SS/RR^ |  |  |  | 1.40 | F7 ^SS/RR^ | 0.020598 |
| *PAC000000001182* | BM501127 | 2 | F7 ^RR/RR^ | F7 ^SS/RR^ | (Q5U1P4) Class III peroxidase 49 precursor (Peroxidase putative) (EC 1.11.1.7), partial (89%) | peroxidase ATP13a | peroxidase 27 precursor, putative, expressed | 1.59 | F7 ^SS/RR^ | 0.05036 |
| *946126A02.y1* | BQ833810 | 2 | F7 ^RR/RR^ | F7  ^RR/SS^ | UP|METK_ORYSA (P46611) S-adenosylmethionine synthetase 1 (Methionine adenosyltransferase 1) (AdoMet synthetase 1) , complete | s-adenosylmethionine synthetase | S-adenosylmethionine synthetase 1, putative, expressed | 1.72 | F7 ^RR/SS^ | 0.014038 |
| *946126A02.y1* |  | 2 | F7 ^RR/RR^ | F7 ^SS/RR^ |  |  |  | 1.69 | F7 ^SS/RR^ | 0.02616 |
| *1091032B12.y1 a* | BQ279334 | 4 | F7 ^SS/SS^ | F7  ^RR/SS^ | UP|METK_HORVU (P50299) S-adenosylmethionine synthetase 1 (Methionine adenosyltransferase 1) (AdoMet synthetase 1) , partial (63%) | s-adenosylmethionine synthetase | S-adenosylmethionine synthetase 1, putative, expressed | 1.86 | F7 ^RR/SS^ | 0.023108 |
| *1091032B12.y1 b* | BQ279334 | 2 | F7 ^RR/RR^ | F7 ^SS/RR^ | UP|METK_HORVU (P50299) S-adenosylmethionine synthetase 1 (Methionine adenosyltransferase 1) (AdoMet synthetase 1) , partial (63%) | putative s-adenosylmethionine synthetase | S-adenosylmethionine synthetase 1, putative, expressed | 1.59 | F7 ^SS/RR^ | 0.006655 |
| *1091032B12.y1 b* |  | 2 | F7 ^RR/RR^ | F7  ^RR/SS^ |  |  |  | 1.57 | F7 ^RR/SS^ | 0.008777 |
| *za72g09.b50* | BE051154 | 2 | F7 ^RR/RR^ | F7 ^SS/RR^ | UP|O49000_ORYSA (O49000) Germin-like protein 4, complete | nectarin - like protein | rhicadhesin receptor precursor, putative, expressed / germin-like protein subfamily 2 member 4 precursor, putative, expressed | 1.44 | F7 ^SS/RR^ | 0.033618 |
| *946063C12.y1* | BE511798 | 5 | F7 ^RR/RR^ | F7  ^RR/SS^ | UP|METK_ORYSA (P46611) S-adenosylmethionine synthetase 1 (Methionine adenosyltransferase 1) (AdoMet synthetase 1) , complete | S-adenosylmethionine synthase 2 | S-adenosylmethionine synthetase 1, putative, expressed / resistance protein, putative | 2.02 | F7 ^RR/RR^ | 0.04668 |
| *exon 1 (eIF3E barley)* |  | 2 | F7 ^RR/RR^ | F7  ^RR/SS^ |  |  |  | 1.49 | F7 ^RR/SS^ | 0.005669 |
| *exon 1 (eIF3E barley)* |  | 2 | F7 ^RR/RR^ | F7 ^SS/RR^ |  |  |  | 1.44 | F7 ^SS/RR^ | 0.021042 |
