# Supplementary material for: Validation of candidate genes putatively associated with resistance to SCMV and MDMV in maize (Zea mays L.) by expression profiling
Source: BMC Plant Biol. 2009 Feb 2;9:15. doi: 10.1186/1471-2229-9-15 (PMC2669481; doi:10.1186/1471-2229-9-15)
Supplement: Additional file 2 — SCMV between-time-point significantly differentially expressed sequences. File 2 illustrates the 28 significantly differentially expressed sequences identified between time points in the SCMV experiment, and gives basic information about the genes retrieved from the analysis. [file 1471-2229-9-15-S2.doc]

| **Gene ID** | **genotype** | **Time point 1** | **Time point(s) 2** | **Gene ID** | **genotype** | **Time point 1** | **Time point(s) 2** |
| --- | --- | --- | --- | --- | --- | --- | --- |
| *605018B03.x1* | F7 | 1 | 2,3,4,5,9 | *MEST24-G11.T3* | F7 | 1 | 3 |
|  |  | 3 | 5 |  |  | 2 | 3 |
|  |  | 4 | 5,6 |  |  | 3 | 9 |
|  | F7 ^RR/RR^ | 1 | 2,3,4,5,9 |  | F7 ^RR/RR^ | 1 | 3 |
|  |  | 2 | 4 |  |  | 3 | 9 |
|  |  | 4 | 9 |  | F7 ^SS/RR^ | 1 | 3 |
|  | F7 ^SS/RR^ | 1 | 2,3,5,9 |  |  | 2 | 3 |
|  |  | 4 | 5,9 |  |  | 3 | 9 |
|  | F7 ^RR/SS^ | 1 | 2,3,4,5,9 |  | F7 ^RR/SS^ | 1 | 3 |
|  |  | 4 | 5,9 |  |  | 2 | 3 |
| *605018B04.x1* | F7 | 1 | 5,9 |  |  | 3 | 5,9 |
|  |  | 3 | 5,9 |  |  | 4 | 9 |
|  |  | 4 | 5,9 | *MEST333-H11.T3* | F7 | 1 | 2,3 |
|  | F7 ^RR/RR^ | 1 | 2,5,9 |  |  | 2 | 4,9 |
|  |  | 2 | 4 |  |  | 3 | 4 |
|  |  | 3 | 9 |  | F7 ^RR/RR^ | 1 | 2,3 |
|  |  | 4 | 5,9 |  |  | 2 | 4,5,9 |
|  | F7 ^SS/RR^ | 1 | 5,9 |  |  | 3 | 4,5,9 |
|  |  | 3 | 5,9 |  | F7 ^SS/RR^ | 1 | 2,3 |
|  |  | 4 | 5,9 |  |  | 2 | 4 |
|  | F7 ^RR/SS^ | 1 | 2,5,9 |  |  | 3 | 4,5,9 |
|  |  | 3 | 5,9 | *MEST40-B08.T3* | F7 | 1 | 3,5 |
|  |  | 4 | 5,9 |  |  | 2 | 3 |
| *606007B06.x1* | F7 | 1 | 2,3,5 |  | F7 ^RR/RR^ | 1 | 3,4,5 |
|  |  | 2 | 9 |  | F7 ^SS/RR^ | 1 | 3,5 |
|  |  | 5 | 9 |  |  | 3 | 9 |
|  | F7 ^RR/RR^ | 1 | 2,3,5 |  | F7 ^RR/SS^ | 1 | 3,4 |
|  |  | 2 | 9 |  |  | 2 | 3 |
|  |  | 3 | 4,9 |  |  | 3 | 9 |
|  |  | 4 | 5 | *MEST40-G05.T3* | F7 | 1 | 5 |
|  |  | 5 | 9 |  |  | 2 | 5 |
|  | F7 ^SS/RR^ | 1 | 2,3,5 |  | F7 ^RR/RR^ | 1 | 5 |
|  |  | 2 | 4,9 |  |  | 2 | 5 |
|  |  | 3 | 4,9 |  | F7 ^SS/RR^ | 1 | 5 |
|  |  | 4 | 5 |  |  | 2 | 5 |
|  |  | 5 | 9 |  | F7 ^RR/SS^ | 1 | 4,5 |
|  | F7 ^RR/SS^ | 1 | 2,3,5 |  |  | 2 | 5 |
|  |  | 3 | 9 | *MEST41-B03.T3* | F7 | 1 | 3,4,5 |
|  |  | 5 | 9 |  |  | 2 | 4,5 |
| *606021F11.x2* | F7 | 1 | 2,3,4,5,9 |  |  | 3 | 4,5 |
|  |  | 2 | 4,5 |  |  | 4 | 9 |
|  |  | 4 | 9 |  |  | 5 | 9 |
|  |  | 5 | 9 |  | F7 ^RR/RR^ | 1 | 3,4,5 |
|  | F7 ^RR/RR^ | 1 | 2,3,4,5,9 |  |  | 2 | 4,5 |
|  |  | 2 | 4,5 |  |  | 3 | 4,5 |
|  |  | 4 | 9 |  |  | 4 | 9 |
|  |  | 5 | 9 |  |  | 5 | 9 |
|  | F7 ^SS/RR^ | 1 | 2,3,4,5,9 |  | F7 ^SS/RR^ | 1 | 3,5 |
|  |  | 2 | 4,5 |  |  | 2 | 4,5 |
|  |  | 4 | 9 |  |  | 3 | 4,5 |
|  |  | 5 | 9 |  |  | 4 | 9 |
|  | F7 ^RR/SS^ | 1 | 2,3,4,5,9 |  |  | 5 | 9 |
|  |  | 2 | 4,5 |  | F7 ^RR/SS^ | 1 | 3,5 |
|  |  | 3 | 5 |  |  | 2 | 4,5 |
|  |  | 4 | 9 |  |  | 3 | 4 |
|  |  | 5 | 9 |  |  | 4 | 9 |
| *614013G06.x1* | F7 | 1 | 2,9 |  |  | 5 | 9 |
|  |  | 2 | 4,9 | *MEST63-E12.T3* | F7 | 1 | 2 |
|  |  | 3 | 9 |  |  | 2 | 4,5,9 |
|  |  | 4 | 9 |  |  | 3 | 4,5,9 |
|  |  | 5 | 9 |  | F7 ^RR/RR^ | 2 | 4,5,9 |
|  | F7 ^RR/RR^ | 1 | 9 |  |  | 3 | 4,5,9 |
|  |  | 2 | 9 |  | F7 ^SS/RR^ | 1 | 2,3 |
|  |  | 3 | 4,9 |  |  | 2 | 4,5,9 |
|  |  | 4 | 9 |  |  | 3 | 4,5,9 |
|  |  | 5 | 9 |  | F7 ^RR/SS^ | 1 | 2,3 |
|  | F7 ^SS/RR^ | 1 | 2,3 |  |  | 2 | 4,5,9 |
|  |  | 2 | 4,9 |  |  | 3 | 4,5 |
|  |  | 3 | 4,9 | *MEST67-A07.T3* | F7 | 1 | 3 |
|  |  | 4 | 9 |  |  | 3 | 9 |
|  |  | 5 | 9 |  | F7 ^RR/RR^ | 1 | 3 |
|  | F7 ^RR/SS^ | 1 | 2,3 |  |  | 3 | 9 |
|  |  | 2 | 4,9 |  | F7 ^SS/RR^ | 1 | 3,4 |
|  |  | 3 | 4,9 |  |  | 3 | 9 |
|  |  | 4 | 9 | *MEST82-F04.T3* | F7 | 1 | 2,3 |
|  |  | 5 | 9 |  |  | 2 | 4,9 |
| *614044F12.x4* | F7 | 1 | 4,5 |  |  | 3 | 4,9 |
|  |  | 2 | 4,5 |  | F7 ^RR/RR^ | 1 | 3 |
|  |  | 3 | 4,5,9 |  |  | 3 | 4,5,9 |
|  | F7 ^RR/RR^ | 1 | 2,3 |  | F7 ^SS/RR^ | 1 | 2,3 |
|  |  | 2 | 4,5,9 |  |  | 2 | 4,9 |
|  |  | 3 | 4,5,9 |  |  | 3 | 4,9 |
|  | F7 ^SS/RR^ | 1 | 3,4,5 |  | F7 ^RR/SS^ | 1 | 2,3 |
|  |  | 2 | 4,5 |  |  | 2 | 4,9 |
|  |  | 3 | 4,5,9 |  |  | 3 | 4,9 |
|  | F7 ^RR/SS^ | 1 | 3 | *Zm06_09h07_R* | F7 | 1 | 3,4 |
|  |  | 2 | 5 |  |  | 2 | 3,4 |
|  |  | 3 | 4,5,9 |  |  | 3 | 5 |
| *945031C10.x1* | F7 ^SS/RR^ | 1 | 2,5 |  | F7 ^RR/RR^ | 1 | 3,9 |
|  |  | 2 | 3 |  | F7 ^SS/RR^ | 2 | 3 |
| *949062B09.y1* | F7 | 1 | 2,5,9 |  | F7 ^RR/SS^ | 1 | 4 |
|  | F7 ^RR/RR^ | 1 | 2,5,9 |  |  | 2 | 3,4 |
|  | F7 ^SS/RR^ | 1 | 2,5,9 | *PAC000000001182* | F7 | 1 | 2,3,4,5,9 |
|  | F7 ^RR/SS^ | 1 | 2,5,9 |  |  | 2 | 5 |
| *MEST12-E11.T3* | F7 | 1 | 4 |  | F7 ^RR/RR^ | 1 | 2,3,4,5,9 |
|  |  | 2 | 4 |  | F7 ^SS/RR^ | 1 | 2,3,4,5,9 |
|  |  | 3 | 5,9 |  |  | 2 | 5,9 |
|  |  | 4 | 5,9 |  | F7 ^RR/SS^ | 1 | 2,3,4,5,9 |
|  | F7 ^RR/RR^ | 1 | 4 |  |  | 2 | 5 |
|  |  | 3 | 5,9 | *946126A02.y1* | F7 | 1 | 2,3,4,5,9 |
|  |  | 4 | 5,9 |  |  | 2 | 9 |
|  | F7 ^SS/RR^ | 2 | 3 |  | F7 ^RR/RR^ | 1 | 2,3,4,5,9 |
|  |  | 3 | 5,9 |  | F7 ^SS/RR^ | 1 | 2,3,4,5,9 |
|  | F7 ^RR/SS^ | 1 | 4 |  |  | 2 | 5,9 |
|  |  | 2 | 3,4 |  | F7 ^RR/SS^ | 1 | 2,3,4,5,9 |
|  |  | 3 | 5,9 |  |  | 2 | 5,9 |
|  |  | 4 | 5,9 | *1091032B12.y1 a* | F7 | 1 | 2,3,4,5,9 |
| *MEST19-G10.T3* | F7 | 2 | 3 |  |  | 2 | 4 |
|  |  | 3 | 9 |  |  | 4 | 9 |
|  | F7 ^RR/RR^ | 1 | 3 |  | F7 ^RR/RR^ | 1 | 2,3,4,5,9 |
|  |  | 2 | 3 |  | F7 ^SS/RR^ | 1 | 3,4,5,9 |
|  |  | 3 | 4,9 |  | F7 ^RR/SS^ | 1 | 2,3,4,5,9 |
|  | F7 ^SS/RR^ | 1 | 3 | *1091032B12.y1 b* | F7 | 1 | 2,3,4,5,9 |
|  |  | 2 | 3 |  |  | 2 | 4,5 |
|  |  | 3 | 5,9 |  | F7 ^RR/RR^ | 1 | 2,3,4,5,9 |
|  | F7 ^RR/SS^ | 1 | 3 |  | F7 ^SS/RR^ | 1 | 2,3,4,5,9 |
|  |  | 2 | 3 |  |  | 2 | 5 |
|  |  | 3 | 5,9 |  | F7 ^RR/SS^ | 1 | 2,3,4,5,9 |
| *MEST22-A03* | F7 | 1 | 3,4,5 |  |  | 2 | 4,5 |
|  |  | 3 | 9 | *za72g09.b50* | F7 | 1 | 2,3,4,5,9 |
|  |  | 4 | 9 |  | F7 ^RR/RR^ | 1 | 2,3,4,5,9 |
|  | F7 ^RR/RR^ | 1 | 3,4,5 |  | F7 ^SS/RR^ | 1 | 2,3,4,5,9 |
|  |  | 3 | 9 |  | F7 ^RR/SS^ | 1 | 2,3,4,5,9 |
|  |  | 4 | 9 | *946063C12.y1* | F7 | 1 | 2,3,4,5,9 |
|  | F7 ^SS/RR^ | 1 | 3,4 |  | F7 ^RR/RR^ | 1 | 2,3,4,5,9 |
|  |  | 3 | 9 |  | F7 ^SS/RR^ | 1 | 2,3,4,5,9 |
|  | F7 ^RR/SS^ | 1 | 3,4 |  |  | 3 | 5 |
|  |  | 3 | 9 |  |  | 4 | 5 |
|  |  | 4 | 9 |  | F7 ^RR/SS^ | 1 | 2,3,4,5,9 |
| *MEST24-E10.T3* | F7 | 1 | 3,4,5 |  |  | 2 | 5 |
|  |  | 2 | 4 |  |  | 3 | 5 |
|  |  | 4 | 9 |  |  | 4 | 5 |
|  | F7 ^RR/RR^ | 1 | 2,3,4,5 |  |  | 5 | 9 |
|  |  | 4 | 9 | *eIF3E barley gene* | F7 | 1 | 2,3,4,5,9 |
|  | F7 ^SS/RR^ | 1 | 3,4,5 |  | F7 ^RR/RR^ | 1 | 2,3,5,9 |
|  |  | 2 | 4 |  |  | 2 | 4 |
|  |  | 4 | 9 |  | F7 ^SS/RR^ | 1 | 3,5 |
|  | F7 ^RR/SS^ | 1 | 3,4,5 |  | F7 ^RR/SS^ | 1 | 3,5 |
|  |  | 2 | 4 |  |  |  |  |
|  |  | 3 | 9 |  |  |  |  |
|  |  | 4 | 9 |  |  |  |  |
|  |  | 5 | 9 |  |  |  |  |
