# Supplementary material for: Validation of candidate genes putatively associated with resistance to SCMV and MDMV in maize (Zea mays L.) by expression profiling
Source: BMC Plant Biol. 2009 Feb 2;9:15. doi: 10.1186/1471-2229-9-15 (PMC2669481; doi:10.1186/1471-2229-9-15)
Supplement: Additional file 5 — MDMV-between-time-point significantly differentially expressed sequences. File 5 illustrates the 2 significantly differentially expressed sequences identified within time points in the MDMV experiment, and gives basic information about the genes retrieved from the analysis. [file 1471-2229-9-15-S5.doc]

| **Gene ID** | **genotype** | **Time point 1** | **Time point(s) 2** |
| --- | --- | --- | --- |
| *605018B04.x1* | F7 | 1 | 9 |
|  |  | 2 | 9 |
|  | F7 ^RR/RR^ | 1 | 3,9 |
|  |  | 2 | 3,9 |
|  |  | 3 | 9 |
| *947026D04.x1* | F7 | 1 | 9 |
|  |  | 2 | 3,9 |
|  | F7 ^RR/RR^ | 1 | 9 |
|  |  | 2 | 3,9 |
|  |  | 3 | 9 |
