# Supplementary material for: Validation of candidate genes putatively associated with resistance to SCMV and MDMV in maize (Zea mays L.) by expression profiling
Source: BMC Plant Biol. 2009 Feb 2;9:15. doi: 10.1186/1471-2229-9-15 (PMC2669481; doi:10.1186/1471-2229-9-15)
Supplement: Additional file 6 — Vectors and primers for insert amplification. File 6 illustrates the 10 different E. coli vectors and their primer sequences utilised in this study for the amplification of inserts to be spotted on the SCMV cDNA microarray. [file 1471-2229-9-15-S6.doc]

| **vector** | **Forward primer (5’ – 3’)** | **Reverse primer (5’ – 3’)** | **Temp. (°C)** |
| --- | --- | --- | --- |
|  |  |  | **For Rev** |
| PBluescript SK (-) | GTA AAA CGA CGG CCA GTG | CAG GAA ACA GCT ATG ACC ATG | 54.3 55.6 |
| PBluescript II SK (+) | GTA ATA CGA CTC ACT ATA GGG CG | CAA TTA ACC CTC ACT AAA GGG | 56.1 54.0 |
| pAD-GAL 4 | AAC TTG CGG GGT TTT TCA | TAC CAC TAC AAT GGA TAT GTA TAT AA | 56.2 55.8 |
| pAD-GAL 4-2.1 | CTA TTC GAT GAT GAA GAT ACC | GAC TAA TAC GAC TCA CTA TAG GGC | 54.6 55.4 |
| pBK-CMV | GTA ATA CGA ACT CAC TAT AGG GCG | CAA TTA ACC CTC ACT AAA GGG | 56.1 54.0 |
| pUC19 | GTA AAA CGA CGG CCA GTG | CAG GAA ACA GCT ATG ACC ATG | 54.3 55.6 |
| pT7T3 PAC | GTA AAA CGA CGG CCA GTG | CAG GAA ACA GCT ATG ACC ATG | 54.3 55.6 |
| pSlip7 | AAT ACG ACT CAC TAT AGG GAG ACA | CAA TTA ACC CTC ACT AAA GGG | 55.0 55.6 |
| pGAD 10 | CTA TTC GAT GAT GAA GAT ACC | GAC TAA TAC GAC TCA CTA TAG GGC | 56.2 55.8 |
| pCMV-Script EX | GTA ATA CGA ACT CAC TAT AGG GCG | CAA TTA ACC CTC ACT AAA GGG | 56.1 54.0 |
