# Supplementary material for: Validation of candidate genes putatively associated with resistance to SCMV and MDMV in maize (Zea mays L.) by expression profiling
Source: BMC Plant Biol. 2009 Feb 2;9:15. doi: 10.1186/1471-2229-9-15 (PMC2669481; doi:10.1186/1471-2229-9-15)
Supplement: Supplementary file 5 [file 1471-2229-9-15-S3.doc]

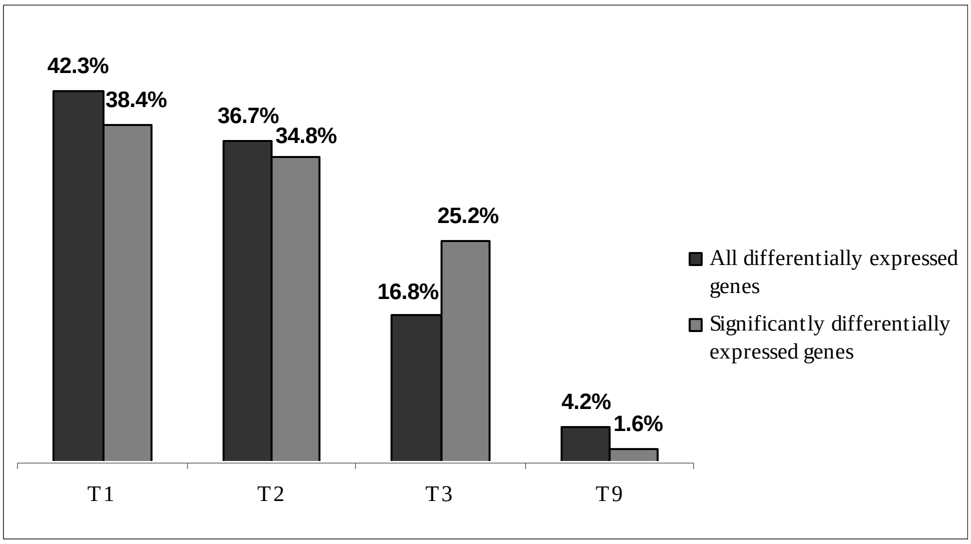
42.3%
38.4%
36.7%
34.8%
16.8%
25.2%
4.2%
1.6%
T1
T2
T3
T9
All differentially expressed genes
Significantly differentially expressed genes
