# Supplementary material for: Validation of candidate genes putatively associated with resistance to SCMV and MDMV in maize (Zea mays L.) by expression profiling
Source: BMC Plant Biol. 2009 Feb 2;9:15. doi: 10.1186/1471-2229-9-15 (PMC2669481; doi:10.1186/1471-2229-9-15)
Supplement: Supplementary file 6 [file 1471-2229-9-15-S4.doc]

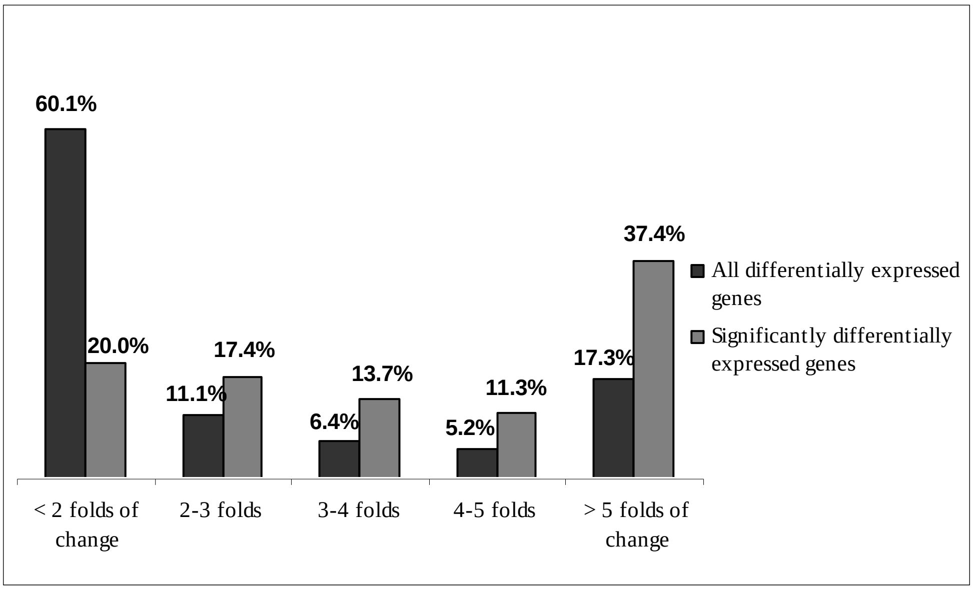
60.1%
20.0%
11.1%
17.4%
6.4%
13.7%
5.2%
11.3%
17.3%
37.4%
All differentially expressed genes
Significantly differentially expressed genes
< 2 folds of change
2-3 folds
3-4 folds
4-5 folds
> 5 folds of change
